# Everolimus exhibits anti-tumorigenic activity in obesity-induced ovarian cancer

**DOI:** 10.18632/oncotarget.7934

**Published:** 2016-03-05

**Authors:** Hui Guo, Yan Zhong, Amanda L. Jackson, Leslie H. Clark, Josh Kilgore, Lu Zhang, Jianjun Han, Xiugui Sheng, Timothy P. Gilliam, Paola A. Gehrig, Chunxiao Zhou, Victoria L. Bae

**Affiliations:** ^1^ Department of Gynecologic Oncology, Shandong Cancer Hospital and Institute, Jinan University, Jinan, P.R. China; ^2^ Division of Gynecologic Oncology, University of North Carolina, Chapel Hill, NC, USA; ^3^ Department of Gynecologic Oncology, Linyi Cancer Hospital, Linyi, P.R. China; ^4^ School of Medicine and Life Sciences, University of Jinan, Shandong Academy of Medical Sciences, Shandong, P.R. China; ^5^ Department of Surgical Oncology, Shandong Cancer Hospital and Institute, Jinan, P.R. China; ^6^ Lineberger Comprehensive Cancer Center, University of North Carolina, Chapel Hill, NC, USA

**Keywords:** ovarian cancer, everolimus, proliferation, mTOR, metabolon

## Abstract

Everolimus inhibits mTOR kinase activity and its downstream targets by acting on mTORC1 and has anti-tumorigenic activity in ovarian cancer. Clinical and epidemiologic data find that obesity is associated with worse outcomes in ovarian cancer. In addition, obesity leads to hyperactivation of the mTOR pathway in epithelial tissues, suggesting that mTOR inhibitors may be a logical choice for treatment in obesity-driven cancers. However, it remains unclear if obesity impacts the effect of everolimus on tumor growth in ovarian cancer. The present study was aimed at evaluating the effects of everolimus on cytotoxicity, cell metabolism, apoptosis, cell cycle, cell stress and invasion in human ovarian cancer cells. A genetically engineered mouse model of serous ovarian cancer fed a high fat diet or low fat diet allowed further investigation into the inter-relationship between everolimus and obesity *in vivo*. Everolimus significantly inhibited cellular proliferation, induced cell cycle G1 arrest and apoptosis, reduced invasion and caused cellular stress *via* inhibition of mTOR pathways *in vitro*. Hypoglycemic conditions enhanced the sensitivity of cells to everolimus through the disruption of glycolysis. Moreover, everolimus was found to inhibit ovarian tumor growth in both obese and lean mice. This reduction coincided with a decrease in expression of Ki-67 and phosphorylated-S6, as well as an increase in cleaved caspase 3 and phosphorylated-AKT. Metabolite profiling revealed that everolimus was able to alter tumor metabolism through different metabolic pathways in the obese and lean mice. Our findings support that everolimus may be a promising therapeutic agent for obesity-driven ovarian cancers.

## INTRODUCTION

Ovarian cancer is the most lethal gynecologic malignancy among women, with an anticipated 21,290 new cases and 14,180 deaths anticipated for 2015 in the United States [1]. Due to the lack of an effective method for the early detection of this disease, most ovarian cancer cases are diagnosed at advanced stages with a survival rate of less than 40% at 5 years [2]. The standard treatment of ovarian cancer is surgical debulking combined with platinum-based combination chemotherapy. Although this therapeutic strategy has high initial response rates, the majority of patients are ultimately faced with a recurrence and chemoresistant disease [3]. Thus, there is a clear need for additional therapeutic options for this deadly disease.

Accumulating epidemiological evidence shows that obesity is associated with an increased risk of developing ovarian cancer compared with women of normal weight [4]. Obesity also confers an increased risk of recurrence, challenges with chemotherapy dosing, and associated poor survival. This results in a 10–17% reduction in overall survival among obese women with ovarian cancer [4–6]. Several mechanisms have been proposed to underlie the relationship between obesity and cancer. Obesity provides a unique adipose tissue microenvironment with concomitant systemic endocrine alterations that favor both carcinogenesis and progression [7]. Elevated levels of sex steroids, activation of the insulin–IGF-1 axis, aberrant productions of adipokines and inflammatory cytokines have all been found to cause tumor growth through activation of the PI3K/AKT/mTOR pathway, including that of ovarian cancers [4, 7, 8]. We have recently found that obesity promotes tumor aggressiveness in a genetically engineered mouse model of serous ovarian cancer [9].

Increased activation of the PI3K/AKT/mTOR signaling pathway as a central regulator of proliferation and metabolism has been implicated in diabetes, obesity and cancer [10]. Amplification of PIK3CA has been observed in approximately 40% of ovarian cancers, and somatic PIK3CA mutations are detected in 18% of ovarian cancers [11, 12]. Phosphorylation of mTOR expression has been found in 55% of ovarian cancers, and high levels of mTOR signaling have been found to be an independent biomarker of poor survival in epithelial ovarian cancer [13, 14]. Moreover, simultaneous AKT and mTOR activation can be present in up to 87% of ovarian tumors [15]. Thus, targeting the PI3K/AKT/mTOR pathway could have a potential role in the treatment of ovarian cancer, particularly for tumors in obese women.

The activation of the mTOR pathway is carried out by two distinct complexes: mTORC1 and mTORC2. mTORC1 is a major downstream target of the PI3K/AKT pathway. It activates protein synthesis by phosphorylating key regulators of messenger RNA translation and ribosome synthesis, which controls the translation of key regulatory proteins [16, 17]. Everolimus is a rapamycin derivative that inhibits the mTOR kinase activity and downstream pathways by acting on mTORC1. It binds to FK506-binding protein-12 forming an inhibitory complex with mTORC1 [18]. The FDA currently has approved everolimus to treat patients with metastatic renal cell carcinoma, advanced pancreatic neuroendocrine tumors and advanced hormone receptor-positive breast cancer [18]. Given that the PI3K/AKT/mTOR pathway is frequently activated in ovarian cancer and obesity, this study aimed to access the anti-tumorigenic efficacy of everolimus in ovarian cancer cells under different glucose conditions *in vitro* and in lean and obese mouse models of serous ovarian cancer *in vivo*.

## RESULTS

### Everolimus inhibits cell proliferation and colony formation in ovarian cancer cells

**Figure 1:**
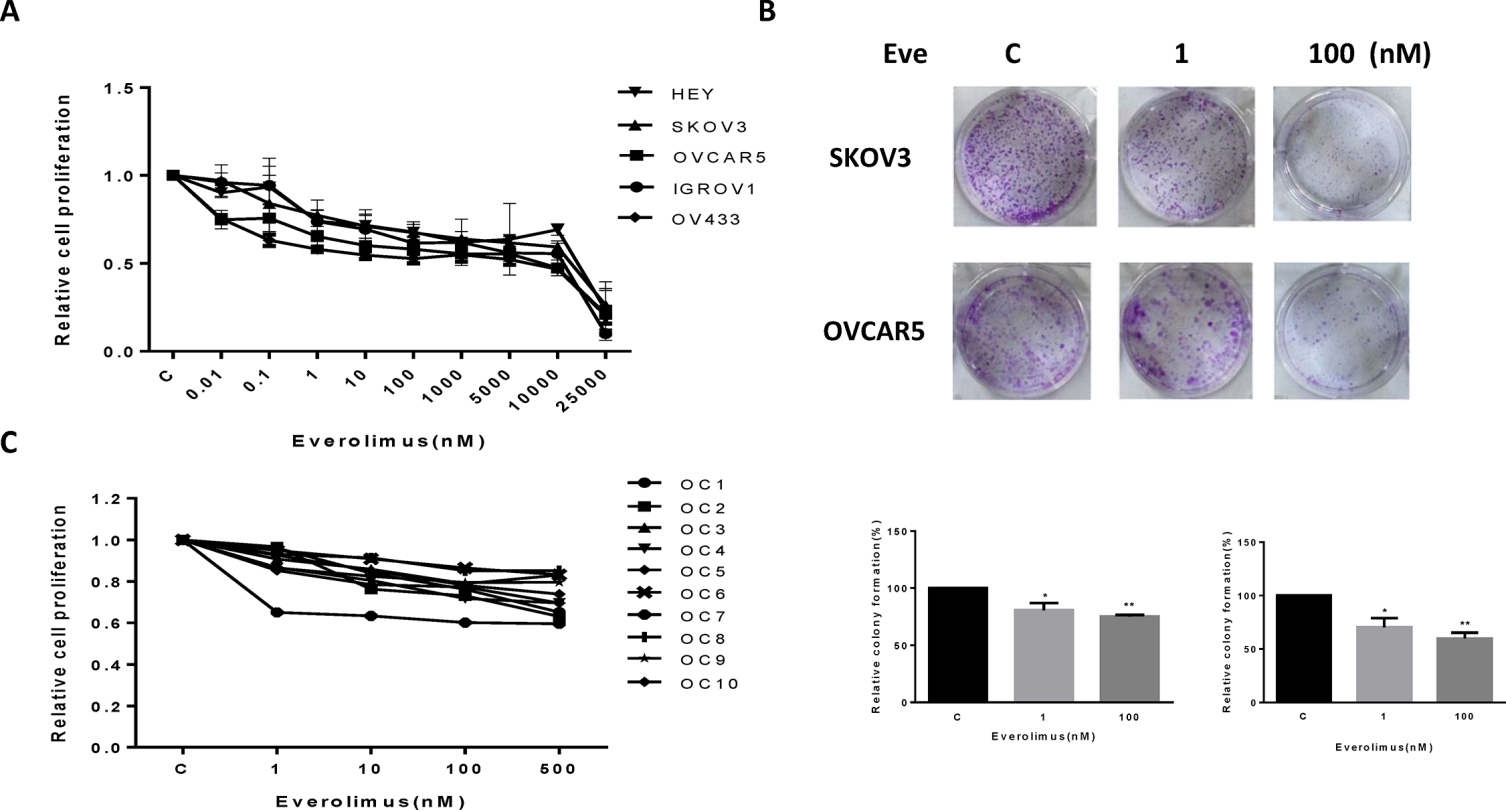
Everolimus suppressed cell proliferation and colony formation The ovarian cancer cell lines, HEY, SKOV3, OVCAR5, IGROV1 and OV433, were cultured for 24 h and then treated with varying concentration of everolimus (from 10 to 25000 nM) in 96 well plates for 72 h. Cell proliferation was assessed by MTT assay (**A**). The effect of everolimus on long term growth in SKOV3 and OVCAR5 was assessed through a colony-forming assay (**B**). Ten primary cultures of human ovarian cancers were cultured for 24 h and treated with everolimus at 10 to 500 nM for 72 h. MTT showed that everolimus decreased cell proliferation in primary cultures of ovarian cancer (**C**). *p < 0.05, **p < 0.01.

The effect of everolimus on growth inhibition was examined in the HEY, SKOV3, OVCAR5, IGROV1 and OV433 cell lines. Using these cell lines, we first examined cell proliferation via MTT assay *in vitro* following 72 hours of exposure to varying doses of everolimus. As shown in Figure 1A, everolimus inhibited cell proliferation in a dose-dependent manner in all five cell lines. We found that everolimus inhibited cell growth even at low doses after 72 hours of treatment, with a mean IC50 between 10 uM to 18 uM. *In vitro* colony formation assays are excellent indicators of long term tumor cell survival and enable predictions of the long term anti-tumor effects of drugs. Given this, we explored whether everolimus had an effect on colonization in the SKOV3 and OVCAR5 cell lines. The results showed that clonogenicity of both cell lines was reduced in a concentration-dependent manner after exposure to everolimus (1 and 100 nM) for 10 days (*p* < 0.05) (Figure 1B).

To further determine the clinical relevance of everolimus treatment, we evaluated the effect of this drug in primary cultures of human ovarian cancer. Ten tissue samples were obtained from patients undergoing surgery for primary epithelial serous ovarian cancer. The primary culture cells were treated with everolimus at varying doses for 72 hours. MTT assays showed that all primary cultures responded to the everolimus treatment with growth inhibition, as seen in the five established ovarian cancer cell lines. None of the primary culture assays reached the point of 50% inhibition at a maximum everolimus dose of 500 nM. Together, these results suggest that everolimus effectively inhibits cell proliferation in ovarian cancer cells *in vitro*.

### Everolimus induces cell cycle G1 arrest and apoptosis in ovarian cancer cells

**Figure 2:**
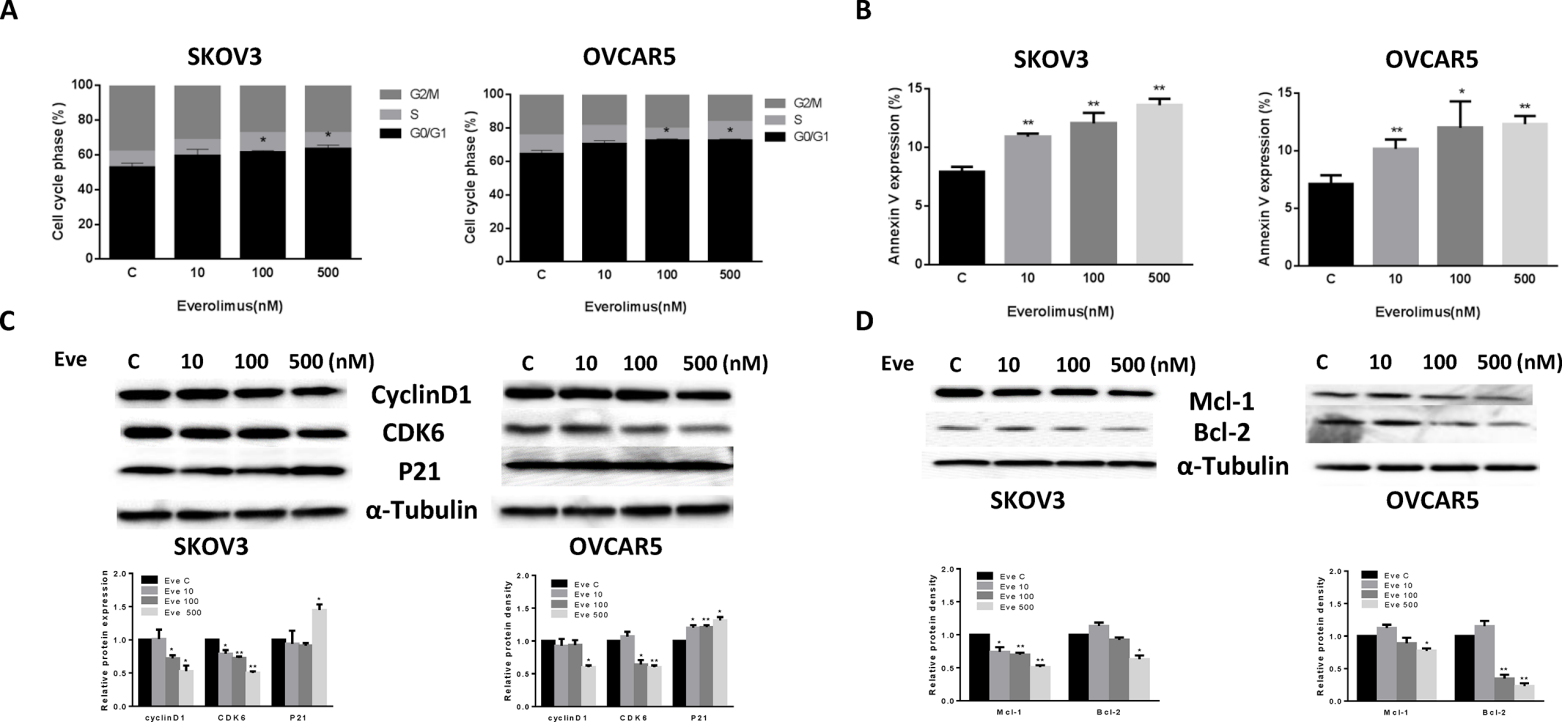
Everolimus induced cell cycle G1 arrest and cellular apoptosis The SKOV3 and OVCAR5 cells were cultured for 24 h and then treated with everolimus at varying doses (from 10 to 500 nM) for 48 h. Cell cycle was examined by Cellometer. Everolimus induced cell cycle G1 arrest in a dose-dependent manner in both cell lines (**A**). The SKOV3 and OVCAR5 cells were treated with varying doses of everolimus for 24 h, and cell apoptosis was examined by an Annexin-V and PI double staining assay via Cellometer. Everolimus significantly increased cell apoptosis in a dose-dependent manner in both cells (**B**). The cells were treated with various concentrations of everolimus as indicated (from 10 to 500 nM) for 24 h, and the expression of cell cycle proteins were assessed using western blotting analysis. Everolimus decreased the levels of cyclin D1 and CDK6 and increased the expression of p21 in the SKOV3 and OVCAR5 cell lines (**C**). The protein expression of Mcl-1 and Bcl-2 was decreased after 24 h of treatment with the indicated doses of everolimus in the SKOV3 and OVCAR5 cells (**D**). *p < 0.05, **p < 0.01.

To evaluate the underlying mechanism of growth inhibition by everolimus, the cell cycle profile was analyzed after treating SKOV3 and OVCAR5 cells with varying doses (10, 100, 500 nM) of everolimus for 24 hours. As shown in Figure 2A, everolimus treatment resulted in cell cycle G1 phase arrest in a dose-dependent manner in both the cell lines when compared with the control groups (*p* < 0.05). To further understand the molecular events underlying the observed G1 arrest, we observed the effects of everolimus on key checkpoint molecules. Everolimus decreased expression of CDK6 and cyclin D1 and increased expression of p21 in both cell lines after 24 hours of treatment (Figure 2C), suggesting that everolimus induces growth inhibition through induction of G1 phase arrest in ovarian cancer cells.

In order to determine whether the reduction of cell viability was due to apoptosis, we detected apoptotic cells by applying an Annexin-V and PI double staining assay using Cellometer. As shown in Figure 2B, everolimus significantly increased Annexin V positive cells of SKOV3 and OVCAR5 in a dose-dependent manner after 24 hours of treatment when compared to the control. In the SKOV3 cells, early apoptosis increased from 8% in control cells to 14.5% in cells treated with everolimus at a dose of 500 nM (*p* = 0.0001). In the OVCAR5 cells, treatment with everolimus enhanced early apoptosis from 6.7% in controls to 12.5% at a dose of 500 nM (*p* = 0.0009). We also found that everolimus reduced protein expression of BCL-2 and MCL-1 in a dose-dependent manner in both cells after treatment for 24 hours (Figure 2D). Together, these results suggest that everolimus not only induces G1 arrest but also causes apoptosis in ovarian cancer cells.

### Everolimus induces cell stress in ovarian cancer cells

**Figure 3:**
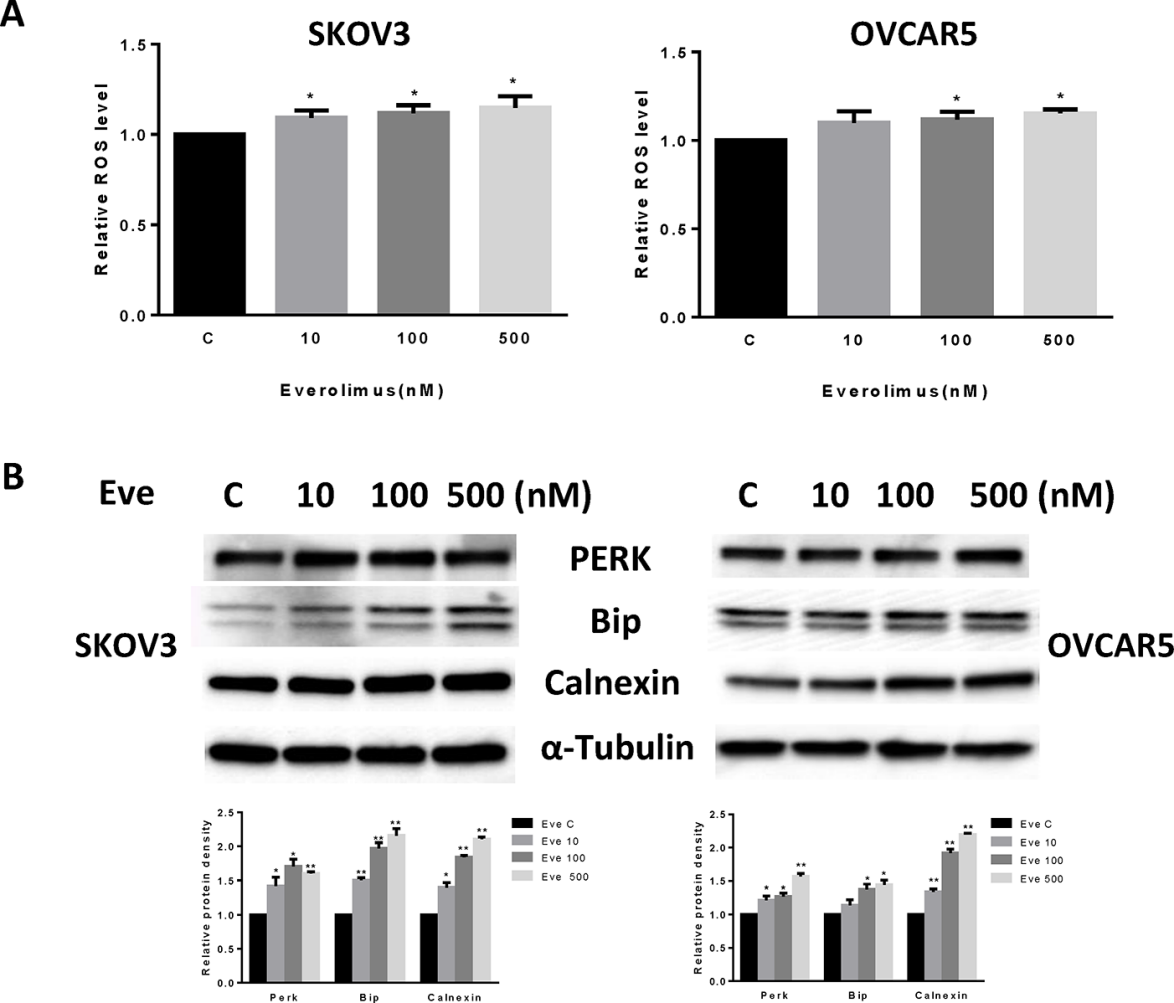
Everolimus induced cell oxidative stress in ovarian cancer cells The SKOV3 and OVCAR5 cells were cultured for 24 h and treated with everolimus at 10 to 500 nM doses for 12 h. ROS production was tested using the DCFH-DA assay. Everolimus increased the ROS level in a dose-dependent manner in the SKOV3 and OVCAR5 cells after 12 h of treatment (**A**). The cells were treated with various concentrations of everolimus for 6 h. Western blotting showed that everolimus increased the expression of stress proteins (PERK, BIP and CALNEXIN) in the SKOV3 and OVCAR5 cell lines (**B**). *p < 0.05.

Given that mTOR activity is strongly stimulated by oxidative stress and inhibited by antioxidants [19], we next investigated the effect of everolimus on induction of cellular stress. We examined the reactive oxygen species (ROS) level and protein expression of cellular stress in both the SKOV3 and OVCAR5 cell lines after treatment with everolimus for 24 hours. Everolimus significantly increased ROS levels in both cell lines in a dose-dependent fashion (*p* < 0.05) (Figure 3A). ROS levels were increased by 14–16% in SKOV3 and OVCAR5 cells at a dose of 500 nM, respectively. Western blotting showed that everolimus also increased the expression of Bip, PREK and Calnexin in a dose-dependent manner after 24 hours of treatment, indicating the cells were under stress (Figure 3B). These results suggest that an increase in ROS production might also be involved in the anti-tumorigenic effects of everolimus in ovarian cancer cells.

### Everolimus inhibits mTOR pathway in ovarian cancer cells

**Figure 4:**
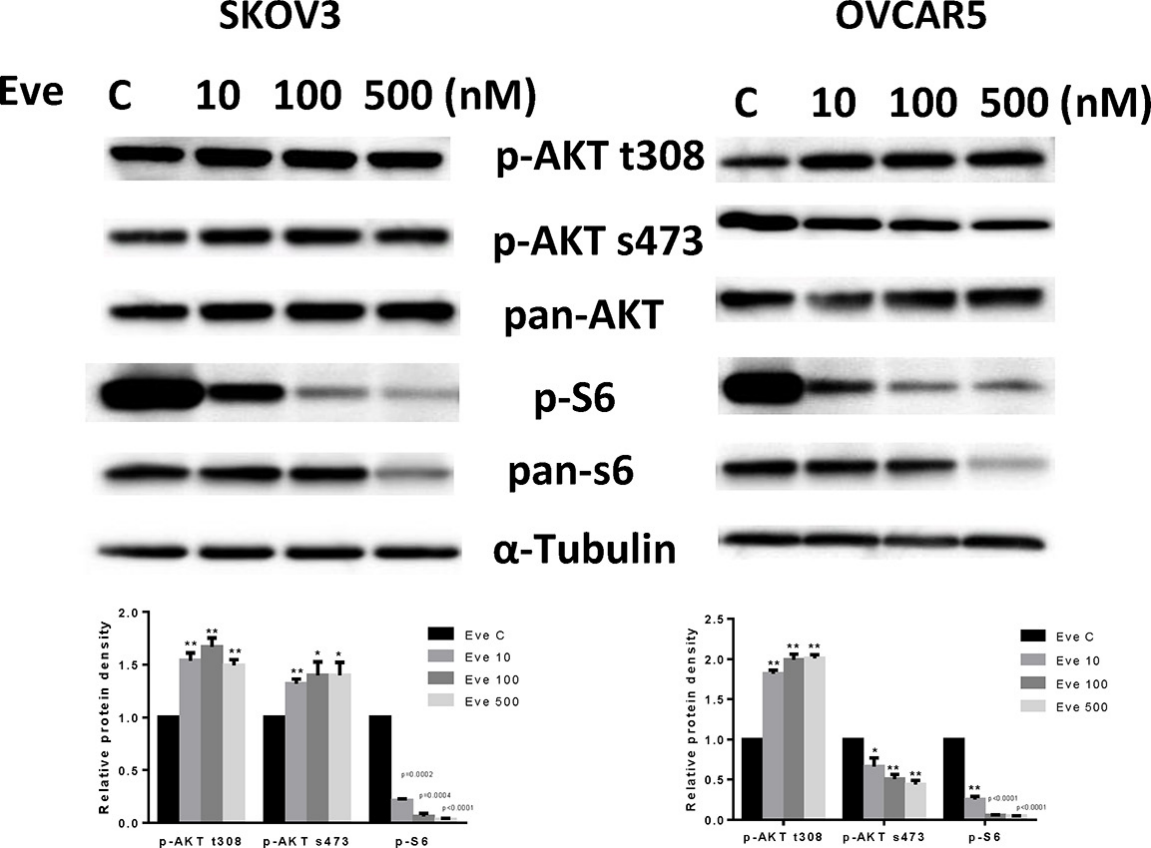
The effect of everolimus on the AKT/mTOR pathway in ovarian cancer cell lines The SKOV3 and OVCAR5 cells were treated with everolimus at different concentrations as indicated (from 10 to 500 nM) for 24 h. Treatment with everolimus reduced the phosphorylated—S6 in a dose-dependent manner. Everolimus increased the expression of phosphorylated-AKT (Thr308 and Ser473) in the SKOV3 cell line, while decreased expression of phosphorylated-AKT (Ser473) was seen in the OVCAR5 cell line (A). *P < 0.05.

To evaluate the effect of everolimus on the mTOR pathway, we treated SKOV3 and OVCAR5 cells with various doses of everolimus for 24 hours. The results showed that phosphorylation of S6 was significantly decreased by everolimus treatment in a dose-dependent fashion in both cell lines, suggesting inhibition of downstream signaling targets of mTOR. Because everolimus is a specific inhibitor of the mTORC1 protein complex and mTORC1 inhibition disrupts the negative feedback loop mediated by PI3K/AKT/mTOR, we assessed changes in phosphorylation of AKT after treatment. As shown in Figure 4, treatment with everolimus effectively increased the phosphorylation of AKT at Thr 308 in both cells and AKT at Ser 473 in SKOV3 cells, indicating that inhibition of mTORC1 by everolimus resulted in an accumulation of phosphorylated AKT activity through a negative feedback loop. Phosphorylation of AKT at Ser 473 was decreased in a dose-dependent manner in OVCAR5 cells in response to everolimus treatment, which suggests everolimus inhibited mTOR signaling through both mTORC1 and mTORC2 signaling pathways in OVCAR5 cells. Our results find that mTORC1 inhibition by everolimus potentially triggers a negative feedback mechanism, and that everolimus may have a function in targeting of mTORC2 complex in ovarian cancer cells [20].

### Glucose affects the sensitivity to everolimus in ovarian cancer cells

**Figure 5:**
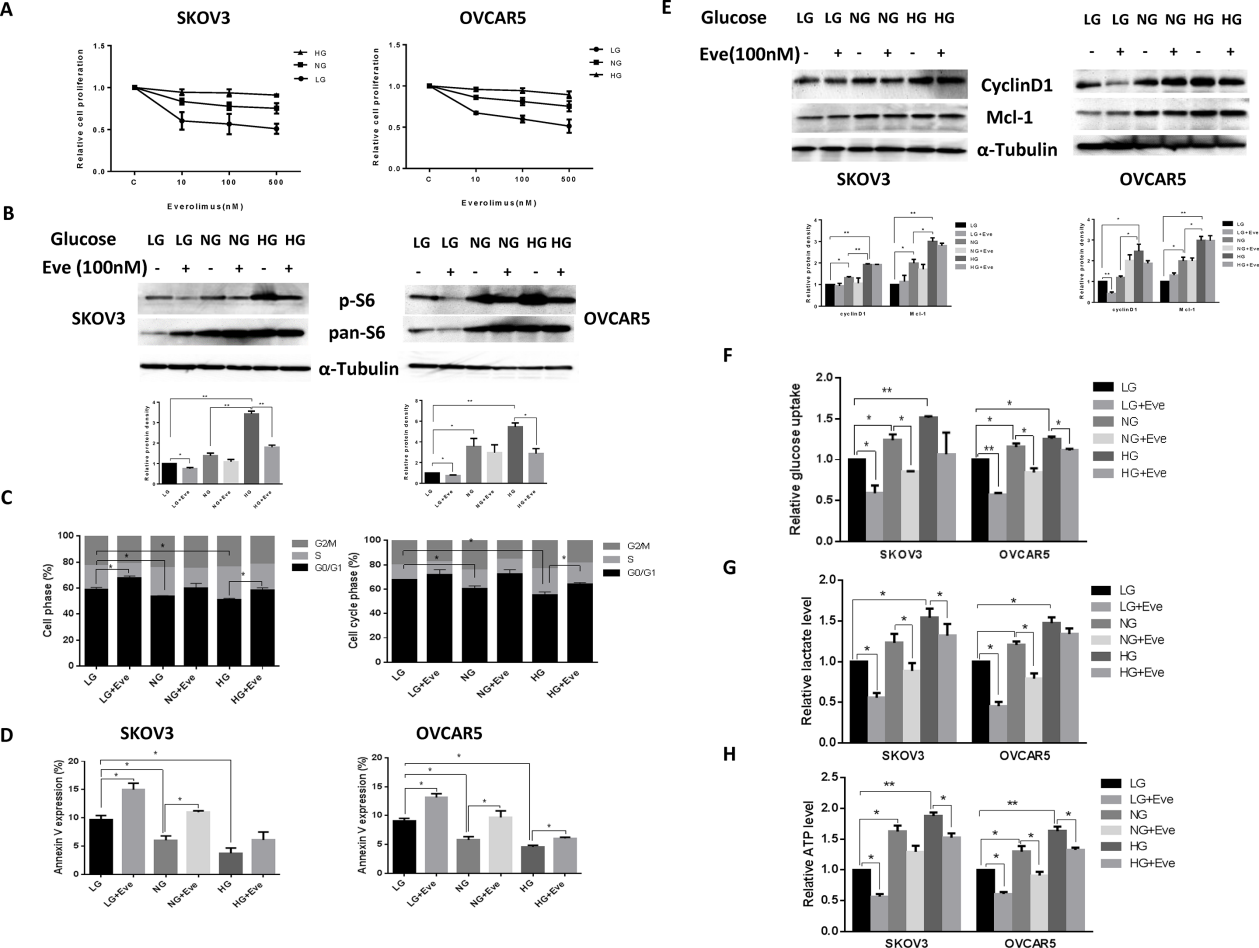
Glucose levels affected the sensitivity to everolimus in ovarian cancer cells The SKOV3 and OVCAR5 cells were cultured in glucose-free media supplemented with 25 mM glucose (HG), 5 mM glucose (NG) and 1 mM glucose (LG) for 24 h and then treated with the indicated concentration of everolimus (from 10 nM to 500 nM) in 96-well plates for 48 h. Cell proliferation was assessed by MTT assay. LG increased the sensitivity to everolimus in inhibition of cell proliferation in both cells (**A**). The cells were treated with evorolimus (100 nM) for 24 h in the media supplemented with varying concentrations of glucose. Western blotting showed that HG obviously increased the expression of phosphorylated-S6 in the SKOV3 and OVCAR5 cells, while everolimus markedly decreased S6 phosphorylation under euglycemic and hypoglycemic conditions (**B**). The analysis of the cell cycle showed that everolimus (100 nM) increased cell cycle phase G1 arrest under euglycemic and hypoglycemic conditions compared with the hyperglycemic condition after 24 h of treatment (**C**). Apoptosis was examined after SKOV3 and OVCAR5 were treated with everolimus (100 nM) at different concentrations of glucose for 24 h, and the results are displayed in graph (**D**). Cyclin D1 and Mcl-1 were determined by Western blotting in the SKOV3 and OVCAR5 cell lines after exposure to everolimus for 12 h with varying concentrations of glucose. HG induced an increase in expression of cyclin D1 and Mcl-1, and everolimus inhibited expression of cyclin D1 and Mcl-1 under euglycemic and hypoglycemic conditions (**E**). The cells were cultured for 24 h and then treated with the everolimus (100 nM) with varying concentrations of glucose for 24 h. The levels of cellular glucose uptake (**F**), cellular ATP and lactate productions (**G** and **H**) were detected. The results showed that hyperglycemia increased the rate of glucose uptake, the production of ATP and lactate levels. Everolimus significantly reduced the rate of glucose uptake, the level of ATP and lactate under all glucose culture conditions. *p < 0.05, **p < 0.01.

Everolimus has been reported to reduce glucose uptake *in vivo* [21–23]. Thus, we examined whether different glucose concentrations influence the effects of everolimus on cell growth in ovarian cancer cells. SKOV3 and OVCAR5 cells were cultured in 96-well plates in their regular media with 25 mM glucose (hyperglycemic condition, HG), 5 mM glucose (euglycemia, NG) and 1 mM glucose (hypoglycemic condition, LG) and then the cells were treated with increasing concentrations of everolimus (from 10 to 500 nM) for 48 hours. MTT results showed that everolimus slightly inhibited cell proliferation in the hyperglycemic condition compared with euglycemia. In contrast, everolimus significantly inhibited cell proliferation in a dose-dependent manner under hypoglycemic conditions in comparison with euglycemic and hyperglycemic conditions (Figure 5A), suggesting that glucose concentration has the ability to affect the sensitivity to everolimus. We next examined the effects of everolimus on S6 protein expression under different concentrations of glucose. The results showed that hyperglycemia caused a three to four fold increase in S6 phosphorylation in both cells compared with hypoglycemia and euglycemia for 24 hours. Everolimus caused a more marked decrease in S6 phosphorylation in euglycemic and hypoglycemic conditions as compared to hyperglycemic conditions (Figure 5B). Everolimus exhibited a significant decrease in inhibition of mTOR/S6 activity for the cells incubated under the hyperglycemic condition, indicating that phosphorylation of S6 may be involved in sensitivity to everolimus under different glucose conditions in ovarian cancer cells.

The effect of everolimus on G1 phase arrest and apoptosis via was further assessed in varying glucose conditions. Incubation of the cells under hypoglycemic conditions for 24 hours increased G1 phase cell cycle arrest from 59% to 65% in SKOV3 cells, and from 67% to 72% in OVCAR5 cells, respectively, compared with euglycemic and hyperglycemic conditions (*p* < 0.05). Treatment with everolimus increased G1 phase arrest in ovarian cancer cells incubated under euglycemic and hypoglycemic conditions compared with hyperglycemia (*p* < 0.05) (Figure 5C). Similar shifts in annexin V expression induced by different concentrations of glucose were seen with SKOV3 and OVCAR5 cells (Figure 5D), suggesting that everolimus attenuated the cell cycle G1 phase and apoptosis under hyperglycemic conditions (*p* < 0.05). Western blotting results showed that hypoglycemia and euglycemic conditions induced a decrease in protein expression of cyclin D1 and MCL-1 in both cells. The expression of cyclin D1 but not MCL-1 was more profoundly influenced by everolimus under hypoglycemic conditions (Figure 5E). These results suggest that everolimus exhibited greater induction of cell cycle G1 arrest and apoptosis under hypoglycemic and euglycemic versus hyperglycemic conditions in ovarian cancer cells.

Cancer cells exhibit enhanced glucose uptake, which in turn increases glycolysis and ATP production ultimately leading to enhanced tumor growth. We examined the effect of everolimus on glucose uptake and ATP levels in ovarian cancer cells incubated with different concentrations of glucose. Both cell lines were treated with everolimus at different concentrations of glucose for 24 hours. The rate of glucose uptake was increased with increasing glucose concentrations, whereas everolimus significantly reduced glucose uptake in each of the treatment groups (*p* < 0.05) (Figure 5F). Similarly, euglycemic and hyperglycemic versus hyperglycemic culture conditions caused a significant increase in the production of ATP and lactate (*p* < 0.05). Everolimus treatment also decreased the levels of ATP and lactate in each treatment groups (*p* < 0.05). However, the cells treated with everolimus under hyperglycemic conditions had less reduction in ATP (Figure 5G and 5H). Together, these results indicate that everolimus effectively inhibits glycolysis and might be more potent in hypoglycemic conditions than in hyperglycemic condition in ovarian cancer cells.

### Everolimus inhibits tumor growth in the KpB serous ovarian cancer mouse model

**Figure 6:**
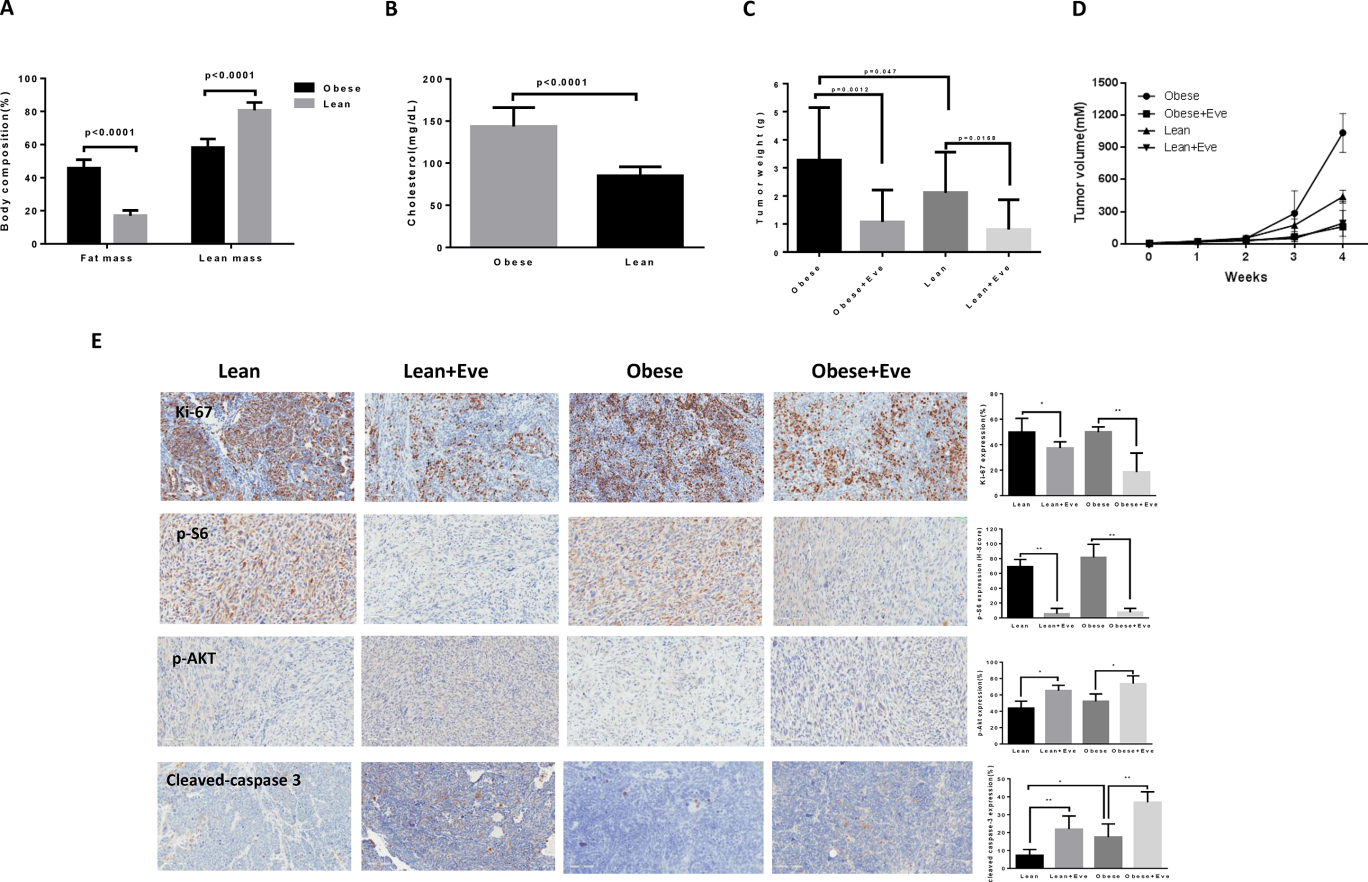
The effect of everolimus on the tumor growth in the KpB serous ovarian cancer mouse model KpB mice were fed high fat diet (HFD) or low fat diet (LFD) at 3 weeks of age to induce obesity. The mice were divided into four groups: obese + placebo, obese + everolimus, lean + placeobo and lean + everolimus. Echo MRI showed that HFD induced an increase in body fat composition in the KpB mice (**A**). The levels of cholesterol were significantly increased in obese mice compared with lean mice (**B**). The obese and lean mice in both groups were treated with everolimus (intraperitoneal injection, 3 mg/kg/day) or placebo. The graphs showed weekly tumor volumes for each group (**C**) and tumor weight after 4 weeks treatment (**D**). The changes of Ki-67, phosphorylated-S6, phosphorylated-AKT and cleaved caspase 3 were assessed by immunohistochemistry in the ovarian cancer tissues. The expression of Ki-67 and phosphorylated-S6 was significantly reduced and cleaved caspase 3 and phosphorylated-AKT (Ser473) was increased in obese group after everolimus treatment compared with the lean group (**E**). *p < 0.05, **p < 0.01.

To further examine whether obesity could affect the anti-tumorigenic potential of everolimus *in vivo*, we used a transgenic serous ovarian cancer mouse model (KpB, K18-gT_121_^+/–^; Brcar1^f/f^; p53^f/f^) fed a high fat diet (HFD) or low fat diet (LFD) at 3 weeks of age to induce obesity [9, 24]. The mice were divided into four groups: obese plus placebo, obese plus everolimus, lean plus placebo, and lean plus everolimus. The initial average body weight of the obese mice when starting treatment was 49.08 gm, while that of lean mice was 30.17 gm (*p* < 0.01, data not shown). EchoMRI showed body composition was significantly altered in obese KpB mice compared to lean controls. Obese mice had a two-fold greater percent body fat and a 20% decrease in percent lean mass (*p* < 0.05) (Figure 6A). The levels of cholesterol were significantly increased in obese mice compared with lean mice (*p* < 0.05) (Figure 6B). However, there was no significant difference in random blood glucose between obese and lean mice over the course of the diet. When the tumor size reached 0.1 × 0.1 cm in diameter by palpation, the mice in both groups were treated with either everolimus (3 mg/kg/day) or placebo (saline) for 4 weeks. During treatment, tumor growth was monitored by palpation twice a week. The mice showed tolerance to everolimus injections and maintained normal activities. Regular twice-weekly measurements yielded no changes in blood glucose, body composition or weight during everolimus or placebo treatment (data not shown). Mice were sacrificed at the end of treatment, and the ovarian tumors were removed, photographed and weighed. Obesity accelerated tumor growth with a 2.5 fold increase in tumor volume and a 40% increase in tumor weight. Both obese and lean mice treated with everolimus had a significant reduction in tumor volume and weight (*p* < 0.05) (Figure 6C and 6D). Ovarian tumor weights were decreased by 66% in obese mice and by 60% in lean mice after treatment with everolimus for 4 weeks when compared with control mice (Figure 6C). These data suggest that obesity promotes ovarian tumor growth and everolimus effectively suppresses the ovarian tumor growth in both obese and lean KpB mice.

To further investigate the anti-tumorigenic activity and mechanism of everolimus *in vivo*, the expression of Ki-67, phosphorylated S6, phosphorylated AKT and cleaved caspase-3 in tumor tissues was evaluated by immunohistochemistry (Figure 6E). As expected, the expression of Ki-67 was significantly reduced following treatment by 64% and 25% compared to controls (*p* < 0.05), in the obese and lean groups, respectively. Everolimus treatment increased cleaved caspase-3 expression from 17% in obese controls to 37% in obese treated mice as compared to 7% of lean controls and 21% in lean treated mice (*p* < 0.05). Consistent with our results *in vitro*, everolimus significantly reduced the expression of phosphorylated S6 and increased the expression of phosphorylated AKT (Ser473) in obese and lean mice compared with the untreated mice, suggesting that everolimus inhibited tumor growth through the mTORC1 pathway *in vivo* (*p* < 0.05). Together, these results further confirm that everolimus inhibits tumor growth of ovarian cancer via targeting of the mTOR/S6 pathway and activation of apoptosis *in vivo*, regardless of obesity status.

### The effect of everolimus on metabolomic profiling in ovarian cancer of obese and lean mice

**Figure 7:**
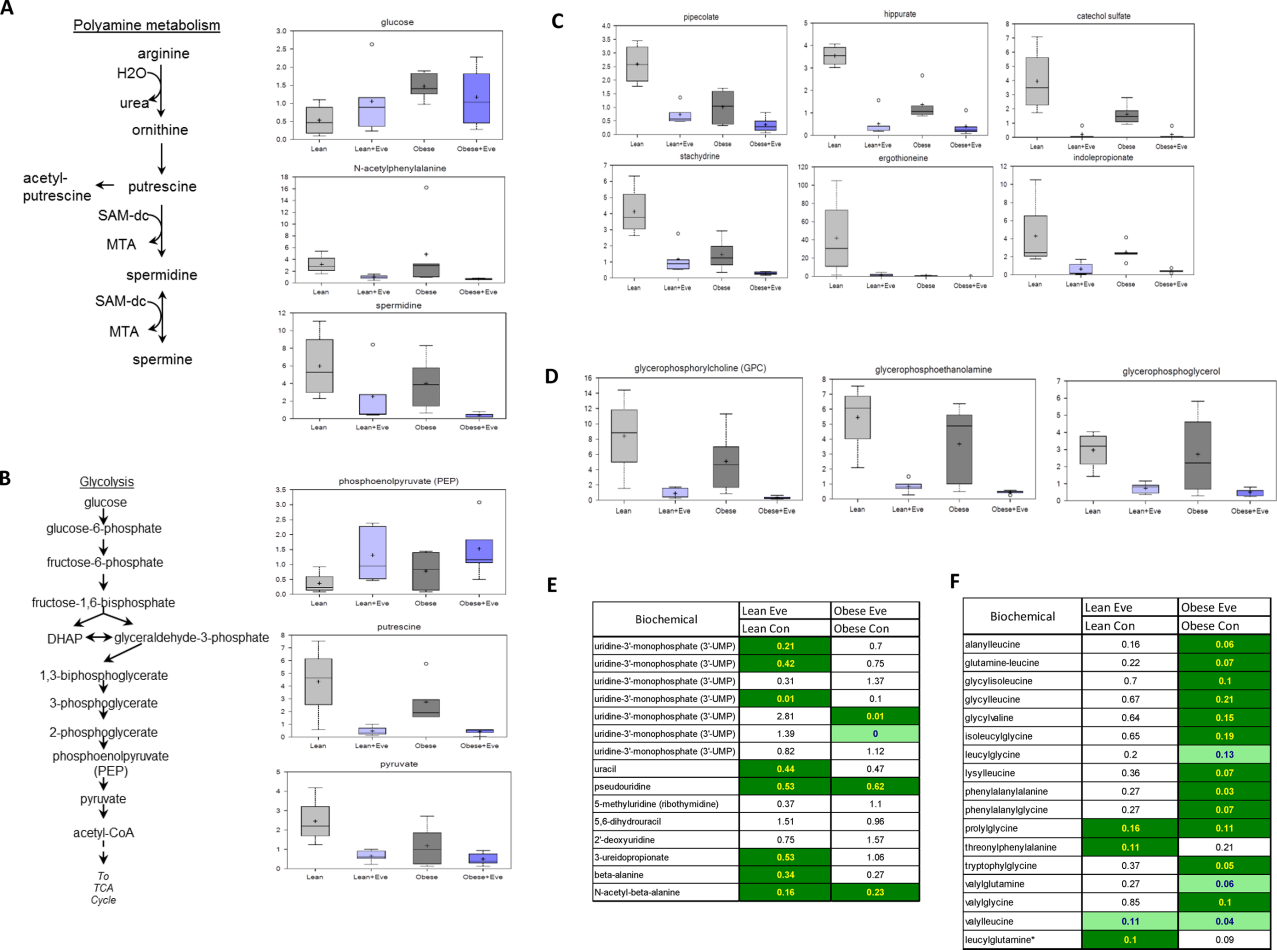
The effect of everolimus on small molecule composition involving metabolites in the KpB mouse model Metabolomic profiling indicated that everolimus down-regulated arginine/putrescine/spermin metabolism in obese and lean KpB mice (**A**). Everolimus inhibited glycolytic activity in obese and lean KpB mice (**B**). The gut microbiome-associated metabolites and compounds were reduced in the lean and obese KpB mice after everolimus treatment (**C**). The glycerophosphocholine (GPC), glycerophosphoethanolamine (GPE), and glycerophosphoglycerol (GPG) were decreased in the obese and lean mice treated with everolimus (**D**). Dipeptide were significantly decreased in obese but not lean mice treated with evorolimus (**E**). The intermediates of pyrimidine metabolism were significantly decreased in the lean mice compared with obese mice after treatment with everolimus (**F**).

We have previously observed significant ovarian tumor progression caused by obesity in the KpB mouse model accompanied by significant differences in metabolomic profiling between obese and lean mice [9] Given this, we sought to determine the effect of everolimus on metabolic pathways associated with cell proliferation in the KpB mouse model using metabolomics. Metabolites involved in polyamine metabolism were down-regulated after treatment with everolimus in both obese and lean KpB mice as compared to control mice, including putrescine, spermidine and N-acetylputrescine (*p* < 0.01). These results suggest that everolimus was able to reduce polyamines needed for packaging newly synthesized DNA into chromatin within the nucleus and are indicative of a decrease of cell proliferation in tumor tissues (Figure 7A). Increased phosphoenolpyruvate (PEP) and decreased pyruvate levels in the glycolytic pathway were observed in obese and lean everolimus-treated mice, suggesting everolimus inhibited glycolysis through inhibition of pyruvate kinase (PK), although glucose levels were 3 fold higher in the tumors of obese versus lean mice (*p* < 0.05) (Figure 7B). Lower levels of gut microbiome-associated metabolites and compounds absorbed from the diet: pipecolate, hippurate, catechol sulfate, stachydrine, ergothioneine and indolepropionate, were detected with everolimus treated obese and lean mice (*p* < 0.05), which could explain how mTOR suppression had systemic effects through alteration of the composition of circulating nutrients in tumor tissues (Figure 7C). The glycerophosphodiesters, glycerophosphocholine (GPC), glycerophosphoethanolamine (GPE) and glycerophosphoglycerol (GPG), were found to be significantly lower in obese and lean mice treated with everolimus (*p* < 0.05) (Figure 7D), reflecting that everolimus may activate glycerophosphodiesterase (s) in tumor tissues. Significant reductions of dipeptides were found in obese but not lean mice treated with everolimus, suggesting everolimus decreases protein degradation to a greater extent in obese mice (Figure 7E). Additionally, significant elevations of intermediates of pyrimidine metabolism were found in lean control mice (Figure 7F), which decreased significantly after everolimus treatment in lean but not obese mice. These metabolomic studies support that everolimus has differential actions depending on whether the ovarian tumors arise from obese or lean mice.

### Everolimus inhibited cell adhesion and invasion in ovarian cancer cells and KpB mice

**Figure 8:**
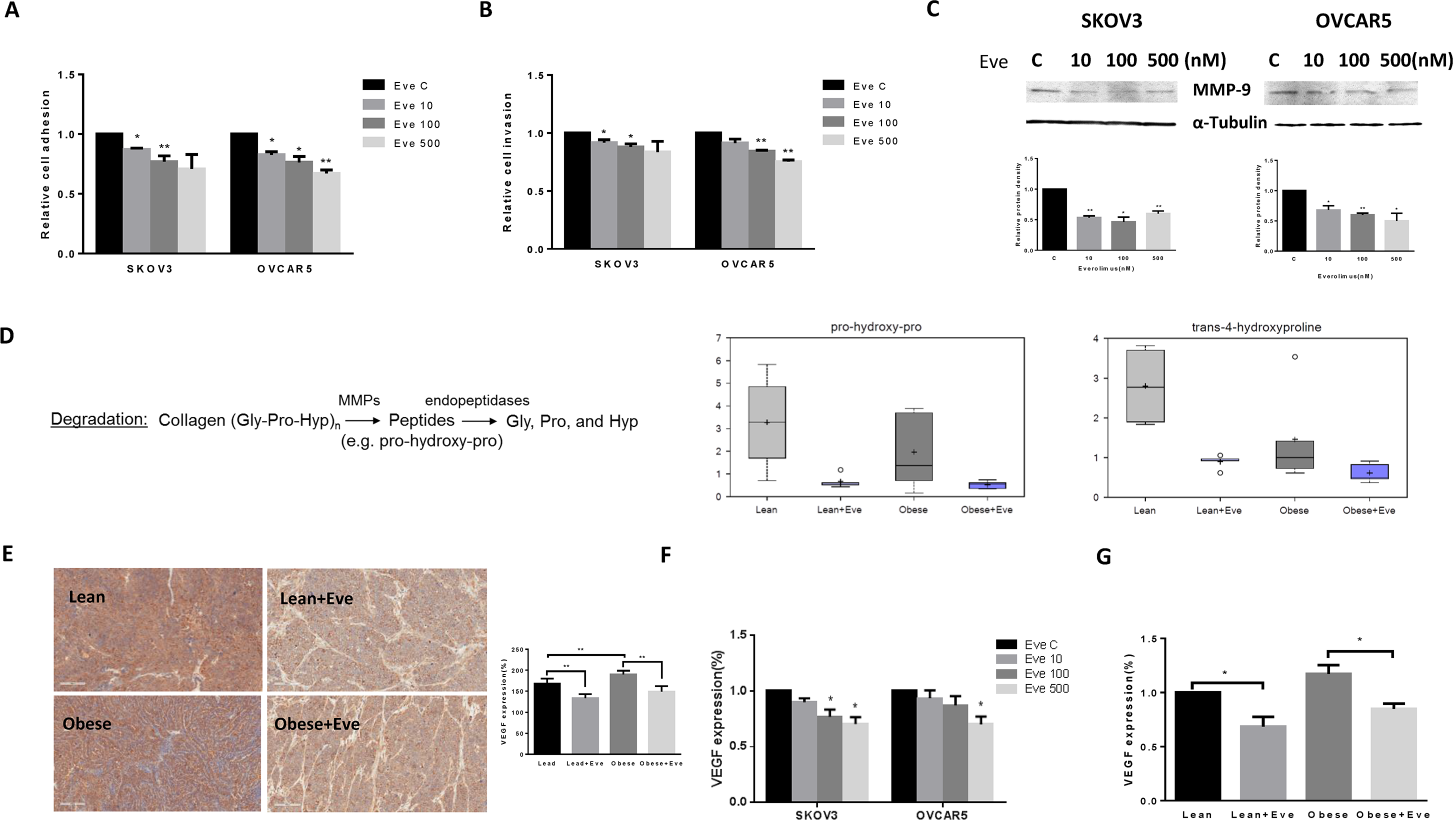
The effect of everolimus on adhesion and invasion in ovarian cancer cells and the obese serous ovarian cancer model The SKOV3 and OVCAR5 cells were cultured for 24 h and then treated with everolimus (100 nM) in a laminin coated 96 well plate or BME coated 96 transwell plate for 24 h or 2 h to asses adhesion and invasion in a plate reader, respectively (**A** and **B**). Everolimus reduced cell adhesion and invasion in both cell lines. The MMP-9 protein was downregulated in the SKOV3 and OVCAR5 cell lines after treatment with everolimus for 24 h (**C**). In the KpB mouse model, metabolomic profiling showed that everolimus induced a degradation of collagen metabolites in obese and lean mice (**D**). Immunohistochemical analysis showed that the expression of VEGF protein was significantly decreased after everolimus treatment in lean and obese mouse (**E**). The level of VEGF after treatment in lean and obese mouse serum was measured by ELISA assays (**F**). SKOV3 and OVCAR5 cells were treated with everolimus at 10 to 500 nM for 24 h. The supernates from culture media were measured by ELISA assay. The results showed that everolimus decreased VEGF production in ovarian cancer cell lines (**H**). *p < 0.05, **p < 0.01.

Cell adhesion and invasion are the basic characteristics of tumor growth and metastasis in ovarian cancer. In order to determine the effect of everolimus on the adhesion and invasion potential of ovarian cancer cells, both laminin adhesion and transwell invasion assays were performed. The SKVO3 and OVCAR5 cell lines were treated with everolimus at varying concentrations for 24 hours for evaluation of cell adhesion and 2 hours for evaluation of invasion. There was significant inhibition of both cell adhesion and invasion in both cell lines (*p* < 0.05) (Figure 8A and 8B). Because the matrix metalloproteinase (MMP) family is involved in the breakdown of extracellular matrix in angiogenesis and metastatic processes, we next evaluated the MMP9 protein expression after 24 hours of everolimus treatment. Everolimus inhibited MMP9 expression in a dose-dependent manner in both cell lines (Figure 8C). Metabolomic profiling demonstrated that everolimus reduced degradation productions of collagen metabolites including pro-hydeoxy-pro and trans-4-hydroxyproline (*p* < 0.05) (Figure 8D), supporting the results of western blotting in the ovarian cancer cell lines. To further confirm the anti-metastatic activity of everolimus *in vivo*, IHC showed that the expression of VEGF was significantly reduced following everolimus treatment in obese and lean mice compared to the controls (*p* < 0.05) (Figure 8E). Similar results were found in (1) mouse serum of obese and lean mice and (2) the culture supernates of ovarian cancer cells treated with everolimus by ELISA assays (Figure 8F and 8G).

## DISCUSSION

The mTOR pathway is a central regulator of various oncogenic processes that contribute to cell proliferation, cell cycle progression, metabolism, angiogenesis and drug resistance in a wide variety of cancers including ovarian cancer [16]. In this study, we describe the anti-tumorigenic efficacy of everolimus attributed to inhibition of mTOR signaling in ovarian cancer, including obesity-driven tumors. Our results showed that treatment with everolimus resulted in a marked reduction in cell growth of ovarian cancer cells, both in established cell lines and primary cultures, which was accompanied by induction of cell cycle arrest, apoptosis and cellular stress. Hyperglycemic conditions increased cell proliferation, and hypoglycemic conditions enhanced the sensitivity to everolimus through disruption of glycolysis. Moreover, everolimus reduced ovarian tumor weight and volume in both obese and lean KpB mice. This reduction in tumor weight and volume coincided with a decrease in expression of Ki-67 and phosphorylated-S6, as well as an increase in cleaved caspase 3 and phosphorylated-AKT, suggesting that everolimus increased AKT activity in *in vivo* models [25]. In addition, inhibition of adhesion and invasion was observed after treatment with everolimus *in vitro* and reduction of VEGF production *in vivo*. Inhibition of mTOR activity by everolimus was effective in altering tumor metabolism with a profound effect on glycolysis, polyamines, dipeptides, glycerophosphodiesters, and nucleotides in both obese and lean KpB mice. These results support that everolimus is a promising anti-tumorigenic agent for the treatment of ovarian cancer, including obesity-driven cancer.

Everolimus has been shown to exhibit potent pre-clinical activities against a wide variety of cancers via induction of cell cycle arrest in G1 phase and apoptosis, including ovarian cancer, small cell lung cancer, renal cell carcinoma, pancreatic cancer and leukemias [17, 26–28]. Through inhibition of mTORC1 function, everolimus blocks essential translational events, including decreases in translation of cyclin D1, c-Myc, hypoxia-inducible transcription factor 1a, ornithine decarboxylase, VEGF and fibroblast growth factor. Ultimately, this results in a decrease of cell proliferation, inhibition of cell cycle progression, alterations in metabolism and induction of apoptosis [17]. Treatment with everolimus has been found to inhibit intra-abdominal dissemination and production of ascites and prolong survival in a xenograft model of ovarian cancer [27]. Everolimus has been also shown to delay tumor onset and progression in a TgMISIIR-TAg transgenic mouse model of ovarian cancer [26]. Our data finds that everolimus inhibited cell proliferation in five ovarian cancer cell lines, as well as ten primary cell cultures of ovarian cancer through induction of cell cycle G1 arrest and apoptosis. Although IC50 values (more than 10 uM) could not be attained with the primary cultures, MTT assays produced nearly flat curves across various concentrations of everolimus from 1 nM to 5 uM in five ovarian cancer cell lines, indicating that low doses of everolimus were effective in inhibition of cell proliferation in ovarian cancer. The concentration of everolimus (1–10 nM) is very close to the therapeutic range recommended (3–8 nM) for everolimus as an immunosuppressant drug at an oral daily dose of 1.5 mg/day. Mabuchi *et al.* report that response to everolimus in mouse models does not correlate with the sensitivity to everolimus *in vitro* in ovarian cancer [26]. Another study also suggests that *in vivo* efficacy of everolimus depended on considerably lower plasma concentrations of everolimus in leukemia [29]. Consistent with this data, our *in vivo* results show that everolimus effectively inhibits tumor growth in a transgenic mouse model of ovarian cancer fed with either HFD or LFD. The precise mechanism to explain this difference in *in vitro* and *in vivo* sensitivity to everolimus remains unknown. It has been proposed that everolimus acts as an allosteric inhibitor *in vitro* resulting in a flat dose–response curve [30].

Obesity is a known risk factor for ovarian cancer, but it is not clear how obesity contributes to carcinogenesis and progression of ovarian cancer. Long term feeding with a HFD in mice initially induces a positive fat balance between fat oxidation and consumption and eventually results in weight gain and fat accumulation [31, 32]. HFD exposure leads to increased levels of cholesterol, triglycerides, adiponectin, proinflammatory cytokines, insulin as well as leptin resistance. These obesity-driven effects subsequently promote the development of cancer, including breast, prostate, liver, and ovarian cancer [33–35]. A recent study showed that exposure to a HFD efficiently activates oncogenic K-ras activity and up-regulates COX2 expression to induce development of pancreatic ductal adenocarcinoma in transgenic mice [36]. High expression of adiponectin in ovarian cancer tissues has an ability to mediate cell proliferation and metabolism [37]. Inflammatory cytokines, such as tumor necrosis factor (TNF)-α, interleukin (IL)-1β and IL-6, produced by a tumor and/or activated by immune cells, have been shown to stimulate cancer cell growth and influence clinical disease status and prognosis [38]. Increased glycolytic metabolism has been observed in ovarian cancer cells, and adipocytes have been found to promote ovarian cancer cell proliferation, migration, expression of cancer associated genes and bio-energetic changes [39, 40]. Additionally, HFD exposure markedly stimulates ovarian tumor growth by up to 6-fold in nude mice compared with controls [41]. The above data all suggests that obesity is associated with the development and progression of ovarian cancer through multiple steps and signaling pathways.

Given that obesity-induced tumor growth involves alterations in AKT/mTOR signaling, everolimus may be a logical choice in obese ovarian cancer patients [42]. In the present study, we induced obesity in the KpB mouse model using a HFD. Mice fed a HFD developed obesity as indicated by increased body weight and fat accumulation, as well as metabolic consequences associated with obesity [32]. The obese mice exhibited much greater tumor volume and tumor size than lean mice. Although everolimus inhibited tumor growth in both obese and lean mice, our *in vitro* results showed that hypoglycemic conditions significantly increased the sensitivity to everolimus. Everolimus showed increased inhibition of cell proliferation through targeting apoptosis, cell cycle arrest and activation of the mTOR/S6 pathway. Given that our mouse model had no difference between serum blood glucose concentrations in obese and lean mice, the effects of the glycemic environment could not be replicated *in vivo*. Because it was observed that phosphorylation of AKT and S6 can be elevated by the hyperglycemic condition in ovarian cancer cells, it is possible that AKT and S6 activities and negative feedback mechanisms are involved in inhibition of cell growth by everolimus at varying glucose concentrations in ovarian cancer cells [16, 25, 43].

Metabolomics offers unbiased identification of subtle changes in small molecule composition (metabolites < 2,000 Da) involving metabolites as affected by signaling pathways and genetic factors, and opens new avenues to investigate the dysregulation of metabolism in cancer biology, cancer diagnosis and chemotherapeutic strategies [44, 45]. Through this approach, we have previously shown that metabolomic profiling exhibited significant differences between ovarian tumors from obese versus lean mice, including metabolically relevant pathways [9]. Similarly, the present metabolic profiling analysis found that ovarian tumors in obese mice have a distinct metabolic signature compared to tumors in lean mice. Everolimus reduced polyamine metabolism required for cell proliferation in obese and lean mice. Recent studies demonstrated that targeting polyamine metabolism has been shown to inhibit the tumor growth *in vitro* and *in vivo*, and cisplatin resistance in ovarian cancer cell lines is associated with polyamine synthesis [46–48]. A major metabolic hallmark of cancer is the frequently elevated glycolytic activity through a shift from oxidative phosphorylation to glycolysis, despite the presence of oxygen, known as the Warburg effect. Inhibition of the mTOR pathway reduces glucose uptake by reducing glucose transporter 1 (GLUT1) expression and/or hexokinase activity [49]. Consistent with previous studies, we found that everolimus significantly inhibited glucose uptake and reduced the production of lactate in ovarian cancer cells. We also found increased PEP and decreased pyruvate in tumor tissues in obese and lean mice, indicating that everolimus inhibited glycolysis through different targets, particularly with pyruvate kinase in the mice [50]. Recent data suggests that the gut microbiota affect nutrient acquisition, energy harvest and the development of obesity and diabetes [51]. Our results found that everolimus, via mTOR suppression, effectively reduced gut microbiome-associated metabolites in both obese and lean tumor tissues. These results suggest that everolimus can decrease gut microbiome-associated metabolites and compounds absorbed from the diet, which may blunt the penetration of these metabolites into ovarian cancer tissues. This could result in a reduced nutrient supply to ovarian cancer for growth. The implications and mechanisms underlying everolimus's metabolic effects on tumor growth are definitely worthy of further exploration.

Three metabolites (glycerophosphodiesters family) associated with lipid metabolism, glycerophosphocholine (GPC), glycerophosphoethanolamine (GPE), and glycerophosphoglycerol (GPG) were significantly reduced with everolimus treatment in obese and lean mice. Glycerophosphodiesters are conserved proteins that contribute to cell proliferation, differentiation and invasion in cancer [52]. GPC is a precursor of phosphatidylcholine (PC), while cleavage of GPC by EDI3 (glycerophosphodiesterase), results in the formation of both choline and glycerol-3-phosphate (G3P) [53], which are closely associated with cell proliferation in cancer [54]. Patients with moderately to poorly differentiated ovarian cancer have higher concentrations of choline in their tumors [55]. Elevated Ki-67, GPC and PC were found in the patients with high-grade prostate cancer [56]. Ovarian cancer tissues showed a significantly decreased GPC and GPE production following *ex vivo* exposure to paclitaxel, cisplatin and carboplatin [55]. Hutschenreuther *et. al*. reported that a higher concentration of GPG is responsible for aerobic glycolysis and enhanced aggressiveness in breast cancer. It is hypothesized that increased GPG provides ATP production, maintains integrity of cellular membranes and participates in signaling including cell migration [57]. These results could indicate that the mTOR pathway plays a role in the regulation of glycerophosphodiesters metabolism. GPC, GPE and GPG may be involved in inhibition of tumor growth induced by everolimus in ovarian cancer. It is not clear whether reduced levels of GPC, GPE, and GPG are the result of less phospholipid breakdown or more.

In addition to its effects on cell growth, everolimus decreased cell adhesion and invasion in ovarian cell lines and VEGF expression in the KpB mice. Metabolomic profiling showed everolimus significantly decreased the degradation products of collagen metabolites in tumor tissues and MMP9 expression in ovarian cancer cells, respectively. This suggests that everolimus has the ability to inhibit the invasive properties of ovarian cancer through suppression of matrix metalloproteinase. Although it remains to be determined if everolimus has other underlying mechanisms to inhibit adhesion and invasion, these results clearly indicate an additional benefit of everolimus for the treatment of ovarian cancer alongside reduction in cell growth. Indeed, the ability to prevent cell invasion could have important clinical implications, given the unique challenges in treating this cancer particularly involving invasion, metastasis, recurrence and drug resistance [2]. Multiple studies have confirmed that everolimus has the potential to inhibit angiogenesis and invasive ability in prostate cancer, esophageal cancer, head and neck cancers, glioma, multiple myeloma, and ovarian cancer [49, 58–62].

The main side effects reported in clinical trials include stomatitis, rash, fatigue, and diarrhea, pulmonary dysfunction, hypercholesterolemia, hypertriglyceridemia, mild leukocytopenia and thrombocytopenia [21, 23, 63]. Hyperglycemia and hypercholesterolemia are other potential metabolic adverse events seen with everolimus treatment. These metabolic changes are associated with the effects of everolimus on mTOR pathway inhibition [64]. These effects are generally mild to moderated in severity, have a low incidence and are clinically manageable [65]. There are several possible mechanisms by which everolimus may cause hyperglycemia and hypercholesterolemia, including impaired insulin-mediated suppression of hepatic glucose production, insulin resistance and reduction of glucose uptake for hyperglycemia and reduction of lipid uptake for hypercholesterolemia [66]. In our study, everolimus treatment did not change the blood glucose or cholesterol levels in either obese or lean mice, suggesting a possible difference between metabolism in humans or mice. Given that the mTOR pathway is highly activated with increased severity of obesity in humans, everolimus may be more successful when used in obese patients with ovarian cancer by reducing mTOR activity. While our *in vivo* results showed that everolimus has equivalent function in obese and lean mice, the significant increase in tumor size seen in the obese mice may account for our inability to detect improved function in obese mice. Regardless, given that everolimus effectively inhibited growth of ovarian cancer in obese mice, everolimus may be a good candidate for ovarian cancer patients with obesity. However, considering everolimus's metabolic adverse events, it may be necessary to optimize levels of lipid and blood glucose before initiating treatment, monitor the changes of blood glucose and cholesterol periodically during the treatment and treat hyperglycemia and hypercholesterolemia according to standard consensus guidelines [23, 63, 66].

Currently, mTOR inhibitors are being studied clinically for use in the treatment of cancers as a single agent or in combination with other chemotherapeutic agents. Several phase I–II clinical trials are now ongoing with mTOR inhibitors in patients with ovarian cancer. A recent critical review indicated that inhibiting a single pathway using mTOR inhibitors may not be sufficient to stop tumor growth; and thus, clinical response may be improved by designing combination therapy protocols. Indeed, tumors respond to mTOR inhibition by creating a negative feedback loop with increased activation of the AKT activity or crosstalk to other signaling pathways to maintain their function, which results in escape from drug-induced growth inhibition [16]. Alternatively, screening for PIK3CA mutations with targeted use of mTOR inhibitors may be another useful strategy since patients with these mutations may be more responsive to PI3K/AKT/mTOR inhibitors than patients without these mutations in gynecologic cancers [3]. Thus, the relationship between clinical response to everolimus and PIK3CA mutations, as well as the role of combination therapy, warrants further investigation in clinical trials in ovarian cancer, including those in obese and lean women.

## MATERIALS AND METHODS

### Cell culture and reagents

The human ovarian cancer cell lines, HEY, SKOV3, OVCAR5, IGROV1 and OV433 were used. The SKOV3 and OVCAR5 cell lines were maintained in DMEM/F12 medium with 5% fetal bovine serum (FBS). The IGROV1 and OV433 cell lines were maintained in RPMI 1640 with 10% FBS. The HEY cell line was maintained in RPMI 1640 with 5% FBS. All medium was supplemented with 100 U/ml of penicillin and 100 ug/ml of streptomycin. The cells were cultured in a humidified 5% CO2 at 37°C. For glucose studies, the SKOV3 and OVCAR5 cell lines were cultured in DMEM medium without glucose (Cat # 11966–025, Gibco), containing 10% dialyzed FBS and supplied with specific concentrations of glucose (1 mM, 5 mM and 25 mM).

Glucose solution, MTT and DMSO were purchased from Sigma-Aldrich (St. Louis, MO). 2-NBDG (2-(N-(7-Nitrobenz-2-oxa-1,3-diazol-4-yl)Amino)-2-Deoxyglucose) was bought from Life Technologies (Grand Island, NY). Enhanced chemiluminescence (ECL) detection reagents were purchased from GE Health care (Piscataway, NJ). The non-fat milk, 20% bull serum albumin (BSA) and RNase A were purchased from Sigma (St. Louis, MO, USA), and all the primary antibodies for phosphorylated-S6, pan-S6, phosphorylated-AKT, pan-AKT, cyclin D1, CDK6, p21, Bcl-2, Mcl-1, Ki-67, VEGF, cleaved caspase 3, perk, bip, calnexin, MMP-9 and α-tubulin were obtained from Cell Signaling Technology (Danvers, MA, USA).

### Cell proliferation assay

The SKOV3 and OVCAR5 cell lines were seeded at 4000 cells/well in 96-well plates in regular media for 24 h. Cells were subsequently treated with varying concentration of everolimus (from 0.01 to 25000 nM) for 72 h. MTT (5 mg/ml) was added to the 96-well plates at 5 μl/well for 1 h. The MTT reaction was terminated through the addition of 100 μl of DMSO. Viable cell densities were determined by measuring absorbance of metabolic conversion of the colorimetric dye at 570 nm. For the glucose experiments, SKOV3 and OVCAR5 cells were seeded at 4000 cells/well in 96-well plates in regular media for 24 h and then treated with different doses of everolimus (from 10 to 500 nm) for 48 h in media supplied with 1, 5 and 25 mM glucose. Cell proliferation was measured by MTT assay. Each experiment was performed in triplicate to assess for consistency of results.

### Colony formation assay

The SKOV3 and OVCAR5 cells growing in log phase were seeded (800 cells/well in a 6-well plate) in their normal growth media. Cells were allowed to adhere for 24 hours, and then treated with everolimus (0, 1 and 100 nM) for 24 hours. Cells were cultured at 37°C for 14 days, with media changes every third or fourth day. Cells were stained with 0.5% crystal violet, and colonies were counted under the microscope. Colony formation assays were performed in duplicate.

### Cell cycle analysis

The SKOV3 and OVCAR5 cells were seeded at 2.5 × 10^5^ cells/well into 6-well plates and incubated overnight, and then treated with everolimus (10, 100 and 500 nM) in regular medium for 48 hours. The cells were harvested and washed with phosphate buffered saline (PBS). The pellet was re-suspended and fixed in 90% pre-chilled methanol and stored overnight at −20°C. The cells were then washed with PBS again and re-suspended in 50 μl RNase A solution (250 μg/ml, 10 mM EDTA) for 30 minutes and then stained with 50 μl of staining solution [containing 2 mg/ml Propidium iodide (Hayward, MA, USA), 0.1 mg/ml Azide (Sigma) and 0.05% Triton X-100 (Sigma)]. The final mixture was incubated for 15 minutes in the dark before analyzing with Cellometer (Nexelom, Lawrence, MA). The results were evaluated using FCS4 express software (Molecular Devices, Sunnyvale, CA). For the glucose experiments, the SKOV3 and OV5 cells were seeded at 2.5 × 10^5^ cells/well into 6-well plates and incubated overnight in regular media, and then treated with 100 nM everolimus for 48 h in media with different concentrations of glucose. Cell cycle was measured by Cellometer. All experiments were performed in triplicate and repeated three times.

### Apoptosis assay

Apoptosis was detected with the Annexin V FITC kit (Biolegend, San Diego, CA) with Cellometer. Briefly, 2 × 10^5^ cells/well were seeded into 6-well plates, incubated overnight and then treated with everolimus (from 0 to 500 nM) in regular medium for 24 h. The cells were then collected, washed with PBS and re-suspended in 100 μl binding buffer. Subsequently, 1 μl of annexin V-FITC (100 ug/ml) and 0.5 uL of propidium iodide (2 mg/ml) were added in the binding buffer and placed in the dark for 15 minutes. The samples were immediately measured by Cellometer. The results were analyzed by FCS4. For the glucose experiments, the SKOV3 and OV5 cells were seeded at 2.5 × 10^5^ cells/well into 6-well plates and incubated overnight in regular media, and then treated with 100 nM everolimus for 24 h in media with different concentrations of glucose. Apoptosis was evaluated using Annexin V FITC kit on the Cellometer. All experiments were performed in triplicate to assess for consistency of response.

### Adhesion assay

Each well in a 96-well plate was coated with 100 μl laminin-1 (10 ug/ml) and incubated at 37°C for 1 h. The fluid was then aspirated and 200 μl blocking buffer was added to each well for 45–60 min at 37°C. The wells were washed with PBS, and the plate was allowed to chill on ice. To each well, 2.5 × 10^3^ cells were added with PBS and varying concentrations of everolimus directly. The plate was then allowed to incubate at 37°C for 2 h. The medium was then aspirated, and the cells were fixed by directly adding 100 μl of 5% glutaraldehyde and incubating for 30 min at room temperature. Adhered cells were then washed with PBS and stained with 100 μl of 0.1% crystal violet for 30 minutes. The cells were washed repeatedly with water, and then 100 μl of 10% acetic acid was added to each well to solubilize the dye. After 5 minutes of shaking, the absorbance was measured at 570 nm, using a microplate reader from Tecan (Morrisville, NC). All experiments were performed in duplicate to assess for consistency of response.

### Invasion assay

Cell invasion assays were performed using 96-well HTS transwells (Corning Life Sciences, Wilmington, NC) coated with 0.5–1X BME (Trevigen, Gaithersburg, Maryland). Starved (serum-free media for 12 h) SKOV3 and OVCAR5 cells (50,000 cells/well) were seeded for 12 h in the upper chambers of the wells in 50 μl FBS-free medium, and the lower chambers were filled with 150 μl regular medium with different concentrations of everolimus. The plate was incubated for 24 h at 37°C to allow invasion into the lower chamber. After washing the upper and lower chambers with PBS once, 100 μl Calcein AM solution was added into the lower chamber and incubated at 37°C for 30–60 minutes. The lower chamber plate was measured by the plate reader for reading fluorescence at EX/EM 485/520 nM. All experiments were performed in duplicate to assess for consistency of response.

### Reactive oxygen species (ROS) assay

ROS generation was assessed using the ROS-sensitive fluorescence indicator, DCFH-DA. To determine intracellular ROS scavenging activity, the SKOV3 and OVCAR5 cells (1.0 × 10^4^ cells/well) were seeded in black 96-well plates. After 24 h, the cells were treated with 10, 100 and 500 nM of everolimus for 12 h to induce ROS generation. After the cells were incubated with DCFH-DA (20 μM) for 30 minutes, the fluorescence intensity was measured at an excitation wavelength of 485 nm and an emission wavelength of 530 nm using a plate reader (Tecan). All experiments were performed in duplicate to assess for consistency of response.

### Glucose uptake assay

The SKOV3 and OVCAR5 cells were seeded into 96-well plates at 4000 cells per well overnight and then were treated with 100 nM everolimus under varying concentrations of glucose for 24 hours. After treatment, the SKOV3 and OV5 cells incubated with 2-NBDG (100 μM) with glucose free medium for 15 minutes. The medium was then replaced with 200 μl HBSS (Life technologies corporation, Grand Island, NY), and the plate was centrifuged for 5 min at 400 rpm. The fluorescence intensity was measured at an excitation wavelength of 485 nm and an emission wavelength of 530 nm using a plate reader from Tecan. Glucose uptake assays were performed in duplicate.

### Measurement of ATP

ATP production was determined using the luminometric ATP assay kit (AAT bioquest, Sunnyvale, CA, USA), following the manufacturer's instructions. In brief, 5 × 10^3^ cells were seeded in 96-well plates and incubated overnight, followed by treatment with everolimus (100 nM) under varying concentrations of glucose for 24 hours. 100 μl/well ATP assay solution was then added, mixed gently and incubated for 20 minutes at room temperature. The luminescence intensity was measured in luminometer mode on a Tecan plate reader. Finally, the ATP levels were normalized based on the viable cell counts measured by the MTT assays. The experiments were performed in triplicate and repeated three times.

### Lactate production assay

Lactate production in the medium was detected using the Lactate Assay Kit (BioVision, Mountain View, CA, USA). Briefly, cells were treated with everolimus (100 nM) for 24 h under varying concentrations of glucose. 2 μl of corresponding media was then removed for the lactate assay. The media was mixed with reaction buffer and incubated for 30 minutes at 37°C in the dark. Fluorescence was then measured at Ex/Em *=* 535/587 nm on a microplate reader. Results were normalized on the basis of the total protein concentration of each sample. All the experiments were performed in triplicate and repeated twice.

### Western blot analysis

Total protein was extracted from the SKOV3 and OVCAR5 cells using RIPA buffer (Boston Bioproducts, Ashland, MA). Protein samples with equal loading (30 ug) were separated by 10–12% SDS-PAGE and transferred onto polyvinylidene fluoride (PVDF) membranes. The membranes were blocked with 5% nonfat milk and then incubated with a 1: 1000 dilution of primary antibodies for overnight at 4°C. The membranes were washed and incubated with a secondary peroxidase-conjugeted antibody for 1 hour at room temperature. The membranes were developed using an enhanced ECL at Alpha Innotech Imaging System (Protein Simple, Santa Clara, CA). After developing, the membranes were re-probed using antibody against α-tubulin to confirm equal loading. The bands' intensity were measured and normalized to α-tubulin. Each experiment was repeated at least twice for consistency of results.

### VEGF assay

To measure VEGF levels, the SKOV3 and OVCAR5 cell lines (2.5 × 10^5^ cells) were plated in 6-well plates and incubated under standard culture conditions overnight. Subsequently, the medium was replaced by serum-free culture medium, and varying concentrations of everolimus were added. The medium was collected after 48 h of exposure to everolimus. 10–50 μl of culture medium was used to measure the levels of VEGF with a VEGF ELISA kit (DVE00, R & D Systems, Minneapolis, MN), according to the manufacturer's instructions. The optical density at 570 nm of each well was measured using a Tecan reader. The VEGF concentration in the serum of mice after exposure to everolimus was measured by the same VEGF ELISA kit.

### Obesity and the K18−gT_121_^+/–^;p53^fl/fl^;Brca1^fl/fl^ mouse model

The K18−gT_121_^+/–^;p53^fl/fl^;Brca1^fl/fl^ (KpB) mouse model is a unique serous ovarian cancer mouse model, wherein the tumor suppressor genes, Brca1 and p53 are specifically and somatically deleted and the retinoblastoma (Rb) proteins are inactivated in the adult ovarian surface epithelium. Inactivation of all 3 Rb proteins by T_121_(a fragment of the SV40 large T antigen) is driven by the keratin 18 (K18) promoter. Expression of the T_121_ transgene and knockout of p53 and Brca1 are conditional and only activated via injection of an adenoviral vector expressing Cre (AdCre) into the ovarian bursa cavity of adult female mice. At approximately 6 months after AdCre injection, tumors develop in the affected ovary, while the un-injected ovary remains normal [24].

For the evaluation of everolimus in the KpB mouse model, 30 mice were provided a high-fat diet (HFD, obese group) (60% kcal from fat, Research Diets, New Brunswick, NJ) to induce obesity and 30 mice were provided a low-fat, control diet (LFD, lean group) (10% kcal from fat, Research Diets, New Brunswick, NJ) ad libitum, beginning at 3 weeks of age. Recombinant adenovirus Ad5-CMV-Cre (AdCre) was purchased from the University of Iowa Transfer Vector Core at a titer of 10^11^−10^12^ infectious particles/ml. AdCre injection occurred at 6–8 weeks to induce ovarian cancer 6 months later (at 8 months of age) [21]. Thirty-six hours following superovulation, the mice were anesthetized, and a single 1 cm incision was made on the dorsal surface of each mouse. The AdCre was then injected via a needle introduced into the oviduct near the infundibulum and into the ovarian bursa, and the incision was closed. All experimental animals were maintained in accordance with the Institutional Animal Care and Use Committee (IACUC) and the NIH guide for the Care and Use of Laboratory Animals. After AdCre injection, each group was randomly divided into control, placebo (saline) group and everolimus treatment group (obese + placebo, obese + everolimus, lean + placebo, lean + everolimus). When tumor size reached 0.1 × 0.1 cm in diameter by palpation, the mice in both groups were treated with everolimus (intraperitoneal injection, 3 mg/kg/day) or placebo. Tumors were palpated and measured by calipers twice a week. After four weeks of everolimus or placebo treatment, mice were euthanized and tumor tissue and blood samples were collected. Tumor volume was calculated using the following equation: tumor volume = (width^2^ × length)/2.

### Immunohistochemical analysis

The mouse tumor tissue was formalin-fixed and paraffin-embedded. Slides (5 μm) were first incubated with Protein Block solution (Dako) for 1 hour and then with the primary antibodies for Ki-67 (1:400), phosphorylated-S6 (1:300), VEGF (1:500), phosphorylated-AKT (1:400) and cleaved caspase 3 (1: 300) for 2 hours at room temperature. The slides were then washed and incubated with appropriate secondary antibodies at room temperature for 1 hour. The slides were washed, and the specific staining was visualized using the Signal Stain Boost IHC Detection Reagent (Cell Signaling Technology), following the manufacturer's instructions. Individual slides were scanned using the Aperio^™^ ScanScope (Aperio Technologies, Vista, CA) and digital images were analyzed for target protein expression using Aperio^™^ ImageScope software.

### Body weight and composition

Prior to starting mice on diet and until sacrifice, body weight was measured weekly. Body composition, including lean mass, fat mass, free water content and total water content, of the obese and lean groups was measured at pre- and post-diet exposures using the EchoMRI-100 quantitative magnetic resonance whole body composition analyzer (Echo Medical Systems, Houston, TX).

### Blood cholesterol

Blood cholesterol from the mice was measured by an automated blood chemical analyzer (Ortho Clinical Diagnostic Inc, Rochester, NY) in the UNC-CH Animal Facility Laboratory, Department of Pathology, UNC, Chapel Hill.

### Metabolomic profiling

Metabolomic profiling was performed on ovarian tumors from control and metformin treated lean and obese mice. Samples were analyzed by Metabolon (Research Triangle Park, NC) according to their standard protocols [67–70]. Briefly, unbiased global metabolomic profiling was achieved using methanol extracts of tumor tissues normalized to serum volume or tissue weight. Analysis of extracts consisted of either ultrahigh performance liquid chromatography (Waters Corporation, Milford, MA) coupled with tandem mass spectrometry (UHPLC/MS/MS; Thermo-Finnigan, San Jose CA) in positive and negative ionization modes, or via gas chromatography/MS analysis (Thermo-Finnigan). Metabolites in tumor tissues were positively identified by matching chromatographic retention time, mass and MS/MS fragmentation patterns to a reference library of over 2500 purified, authenticated biochemicals. Data are presented as relative measures of “scaled intensity” and median scaling to 1. Missing values were imputed with the minimum.

Gas chromatography time-of-flight mass spectrometry (GC-TOFMS, Leco Corporation, St Joseph, MI) and liquid chromatography coupled with time-of-flight mass spectrometry(LC-TOFMS, Agilent Corporation, Santa Clara, CA) were used to analyze tumors from the four groups (*N* = 5/group) at Metabolon Inc (Durham, NC, USA) following our previous publication [9]. Metabolite annotation was performed by comparing the mass spectrum and retention time to an in-house library and NIST library (GC-TOMFS) or HMDB (LC-TOFMS). Compounds were identified by comparison to library entries of purified standards or recurrent unknown entities. Identification of known chemical entities was based on comparison to metabolomic library entries of purified standards based on chromatographic properties and mass spectra.

### Ovarian cancer tissue samples collection and primary cell culture

Ten tumor specimens were sampled from patients undergoing surgery for primary serous ovarian carcinoma at the University of North Carolina at Chapel Hill (UNC-CH). The protocol was reviewed and approved by the Institutional Review Board at UNC-CH. Tumors were staged and graded according to the criteria of the International Federation of Obstetrics and Gynecology (FIGO, 2009). For the primary cultures of human ovarian cancer cells, the freshly obtained tissues were washed three times with Hank's Buffered Salt Solution (HBSS), and then gently minced by scissors in DMEM/F12 medium containing 10% fetal bovine serum (FBS). These tissues were then digested in 0.2% collagenase IA, 100 U/ml penicillin and streptomycin for 30–60 minutes at 37°C water bath with shaking. After two centrifugations with PBS solution, cells were re-suspended and diluted to 1 × 10^5^ cells/ml with DMEM/F12 medium. Subsequently, 1 × 10^4^ cells/well were seeded into 96-well plates for 24 h, and then treated with everolimus as indicated for 72 h. Cell proliferation was measured by MTT assay.

### Statistical analysis

Results were compared by Student's *t* test and data were expressed as mean ± S.E. Statistical significance was defined to be *p* < 0.05.
